# Oxidative Stress Responses and Nutrient Starvation in MCHM Treated *Saccharomyces cerevisiae*

**DOI:** 10.1534/g3.120.401661

**Published:** 2020-10-27

**Authors:** Michael C. Ayers, Zachary N. Sherman, Jennifer E. G. Gallagher

**Affiliations:** Department of Biology, West Virginia University, Morgantown, WV 26506

**Keywords:** environmental stress response, MCHM, *Saccharomyces cerevisiae*, genetic screen, reactive oxygen species, nutrient starvation, aromatic amino acids, tryptophan

## Abstract

In 2014, the coal cleaning chemical 4-methylcyclohexane methanol (MCHM) spilled into the water supply for 300,000 West Virginians. Initial toxicology tests showed relatively mild results, but the underlying effects on cellular biology were underexplored. Treated wildtype yeast cells grew poorly, but there was only a small decrease in cell viability. Cell cycle analysis revealed an absence of cells in S phase within thirty minutes of treatment. Cells accumulated in G1 over a six-hour time course, indicating arrest instead of death. A genetic screen of the haploid knockout collection revealed 329 high confidence genes required for optimal growth in MCHM. These genes encode three major cell processes: mitochondrial gene expression/translation, the vacuolar ATPase, and aromatic amino acid biosynthesis. The transcriptome showed an upregulation of pleiotropic drug response genes and amino acid biosynthetic genes and downregulation in ribosome biosynthesis. Analysis of these datasets pointed to environmental stress response activation upon treatment. Overlap in datasets included the aromatic amino acid genes *ARO1*, *ARO3*, and four of the five *TRP* genes. This implicated nutrient deprivation as the signal for stress response. Excess supplementation of nutrients and amino acids did not improve growth on MCHM, so the source of nutrient deprivation signal is still unclear. Reactive oxygen species and DNA damage were directly detected with MCHM treatment, but timepoints showed these accumulated slower than cells arrested. We propose that wildtype cells arrest from nutrient deprivation and survive, accumulating oxidative damage through the implementation of robust environmental stress responses.

The chemical 4-methylcyclohexane methanol (MCHM) was a previously little-studied chemical involved in the processing of coal, until a rusted storage tank resulted in a spill of crude MCHM into the Elk River near Charleston, WV. The spill’s size and location adjacent to a drinking water treatment intake were sufficient to fill homes with an overpowering odor that left many fearful of health consequences (Thomasson *et al.*, 2017). This spill interrupted the water supply of approximately 300,000 residents. Research on MCHM since the Elk River spill has increased dramatically over concerns about the lack of characterized physical and biological properties of this chemical (Weidhaas *et al.*, 2016). For instance, an improved toxicological study on MCHM’s effect on model organism viability has been performed (West Virginia Chemical Spill: Collective NTP Findings and Supporting Files, n.d.), as well as one study on the potential stress responses it may produce in yeast (Lan *et al.*, 2015). However, these studies had a focus on a few specific toxicological outcomes and predetermined stress pathways that may miss other cellular changes. Recently, research has been begun to identify the biochemical and transcriptional changes that MCHM may produce in organisms (Pupo *et al.*, 2019a; Pupo *et al.*, 2019b).

The environmental stress response (ESR) is a shared transcriptional response to multiple stressors, including heat shock, osmotic shock, and hydrogen peroxide treatment, among others (Gasch *et al.*, 2000). The role of paralogous transcription factors Msn2 and Msn4 are important for a large portion of the ESR transcriptional induction (Gorner *et al.*, 1998; Martínez-Pastor *et al.*, 1996). The transcriptional programming of the environmental stress response incorporates signals from diverse stress pathways including nutrient starvation (TOR), osmolarity stress (HOG), and others, into the Msn2/4 transcriptional activators (Capaldi *et al.*, 2008; De Wever *et al.*, 2005; Garmendia-Torres *et al.*, 2007; Gorner *et al.*, 1998; Gutin *et al.*, 2015; Santhanam *et al.*, 2004). Implementation of the stress response involves two waves of Msn2/4 import to the nucleus that modulate the initial intensity and prolonged duration of the response (Gutin *et al.*, 2019). The signaling kinase Mck1 is indispensable for the prolonged response requiring the second import of active Msn2/4 to the nucleus (Gutin *et al.*, 2019), although it is unclear how the signal is maintained as there does not appear to be direct Mck1 interaction with Msn2 (Hirata *et al.*, 2002). The karyopherin Msn5 involved in nuclear import and export activity is important to maintain the prolonged stress response activity of Msn2/4 in the ESR, apparently through the export of initial wave Msn2 from the nucleus, allowing for the second wave of transcriptional activators to function (Gutin *et al.*, 2019).

Amino acid biosynthesis is predominately regulated by the general amino acid transcriptional activator *Gcn4*. Under amino acid starvation, the levels of *Gcn4* increase due to the decreased degradation and increased translation of the protein (reviewed in Hinnebusch 1997; Meimoun *et al.*, 2000). *Gcn4* binds to a consensus promoter sequence upstream of many of the amino acid biosynthetic genes to positively regulate their expression (Arndt & Fink 1986; Hill *et al.*, 1986; Oliphant *et al.*, 1989). This amino acid transcriptional control includes the aromatic amino acid biosynthetic gene *ARO3* and the specific tryptophan biosynthetic genes *TRP2**, **TRP3**, **TRP4*, and *TRP5* (Braus 1991). Tryptophan has been implicated for roles in stress tolerances other than nutrient starvation. SDS sensitivity, a cell wall and plasma membrane integrity stress that involves Mck1 effectors in yeast, is dependent on tryptophan biosynthesis and levels of tryptophan and tyrosine in the media and cell (Schroeder & Ikui 2019). Tryptophan biosynthetic mutants, *trp1**-**5*, were also reported in a special warning for their use as auxotrophic markers in yeast genetics due to an aberrant sensitivity to many stressors including rapamycin, high pH, and several metal cations (González *et al.*, 2008). A more recent report has shown that tryptophan depletion due to a combination of transporter dysfunction at low temperature and *trp1**-**5* mutants also confers sensitivity to the DNA damaging agents MMS and HU (Godin *et al.*, 2016). The role of the aromatic amino acids and their biosynthetic pathways in stress tolerance other than nutrient starvation is not fully understood, but it is well established.

Reactive oxygen species (ROS) have previously been implicated as a source of toxicity in cells treated with MCHM (Lan *et al.*, 2015). Cells contain conserved robust networks to mitigate the toxic effects of ROS. These include various proteins, from enzymes that detoxify the reactive species directly, to proteins that repair damage within the cell, such as to DNA (Ayer *et al.*, 2014). The thioredoxin and glutathione (GSH) pathways have significant roles in the cell’s response to ROS. They perform overlapping functions reducing thiol oxidation that can damage proteins in the cytosol. Furthermore, GSH has roles in iron homeostasis between the mitochondria and vacuole, and potentially as a possible buffer for oxidation in disulfide bond formation during protein folding in the ER (Cuozzo & Kaiser 1999; reviewed by Toledano *et al.*, 2013). Mitochondria serve as a major producer of ROS in the cell as oxidative phosphorylation leaks electrons to molecular oxygen to produce superoxide anions, so these pathways are activated by normal cellular metabolism (reviewed by all the following, Ayer *et al.*, 2014; Perrone *et al.*, 2008; Temple *et al.*, 2005). However, they also become important during the response to toxic chemicals, which can produce ROS directly, or else indirectly through metabolism and attempted detoxification in the vacuole. Toxicity of chemicals that produce ROS in the cell may be mitigated through treatment with antioxidants or intensified through damage to the cellular stress networks (Couto *et al.*, 2016; Sekito *et al.*, 2014).

Much of a yeast cell’s response to stress involves the vacuole (Li & Kane 2010). This organelle serves as a site for various processes of degradation, detoxification, and metal ion and pH homeostasis (Li & Kane 2010). The vacuolar ATPase (v-ATPase) is a structure in yeast that is highly conserved and has been adapted to perform a wide range of functions in various eukaryotes. In many animals, including *Drosophila*, homologs of the v-ATPase are known to contribute to nerve function via vesicular excretion of neurotransmitters in animals including humans (Hiesinger *et al.*, 2005). In yeast, the v-ATPase is responsible for acidifying the vacuole interior, creating a proton gradient that is responsible for multiple homeostatic processes (reviewed by the following, N. Nelson & Harvey 1999; Nishi & Forgac 2002). As such, its effects on metal ion transport and vacuolar acidification result in a set of knockout phenotypes for many of the v-ATPase’s subunits (Hemenway *et al.*, 1995; H. Nelson & Nelson 1990; Ohya *et al.*, 1991; Sambade *et al.*, 2005). This *vma*- phenotype includes sensitivities to metal ions, reactive oxygen species, and pH perturbations in either direction. Inositol depletion through mutation or chemical treatment negatively impacts the assembly and activity of the v-ATPase in yeast (Deranieh *et al.*, 2015; Ohya *et al.*, 1991). Any chemical that can inhibit or damage the v-ATPase would likely have distinct consequences for the cell’s ability to cope with other stresses.

The goal of this study was to characterize the response of yeast cells to the foreign chemical MCHM. Treatment of yeast with MCHM created pleiotropic effects on networks throughout the cell. We employed methods including viability assays, RNAseq, flow cytometry, and a genetic screen of knockout strains to characterize these effects. We found several expected changes to networks, such as the pleiotropic drug response ABC transporters that remove xenobiotics from the cell. Our data show direct evidence of ROS and DNA damage following MCHM treatment. The stress also revealed a role for the aromatic amino acid biosynthetic pathway outside nutrient availability for the response to this particular stressor. Any toxicity that these cellular changes caused did not result in large changes in cellular viability but instead resulted in the arrest of the cell cycle in G1. Effects on the cell were wide-ranging, but wildtype cells were able to implement the ESR to recover from exposure at levels higher than spill levels.

## Materials and Methods

### Yeast strains and media

The haploid BY4741 strain (MATA, *his3**Δ, **leu2**Δ, **ura3**Δ, **met15**Δ)* (Brachmann *et al.*, 1998) and its MATalpha counterpart BY4742 (*his3**Δ, **leu2**Δ, **ura3**Δ, **lys2**Δ*), were used for the majority of experiments as denoted. RNAseq, viability, growth, comet and flow cytometry assays used the BY4741 strain. The genetic screen used the BY4742 collection (Giaever *et al.*, 2002). The YJM789 wildtype strain (MATalpha, *lys2*) and its previously generated *aro1* knockout (MATa, *ARO1*::NAT^R^)(Rong-Mullins *et al.*, 2017a) were used in serial dilution growth assays only as shown. Rich media containing 1% yeast extract, 2% peptone, and 2% dextrose (YPD) or minimal media containing 0.67% yeast nitrogen base and 2% dextrose (YM) with histidine, leucine, uracil, and methionine supplementation for BY4741 strains were used in the various experiments as indicated. Experiments where the aromatic amino acids were supplemented in excess to YPD media were done to a final concentration of 0.02mg/mL tryptophan, 0.03mg/mL tyrosine, and 0.05mg/mL phenylalanine. The strains used in the *TAT2* overexpression serial dilution assay were created by transforming BY4742 yeast with either pRS315 (*CEN, **LEU2*) plasmid (Sikorski & Hieter 1989) or commercially available *LEU2**+* multicopy yeast genomic tiling collection plasmids containing genomic regions from chromosome XV corresponding to the region surrounding the *TAT2* ORF (Jones *et al.*, 2008). Supplementations of MCHM, drugs, or other nutrients were added to YPD or YM media as indicated for each experiment. MCHM for experiments is the same crude MCHM formulation that made up the primary chemical presence in the Elk River spill. This formulation is approximately 89% 4-methylcyclohexane methanol, with other cyclohexanes making up the rest of the material was obtained directly from the manufacturer, Eastman Chemical Copmany (Kingsport, TN, US). All concentrations of MCHM indicated in figures are in ppm of crude MCHM formulation, so all conclusions are for this formulation, not pure MCHM.

### Growth and viability assays

Growth and viability assays were performed as previously described (Rong-Mullins *et al.*, 2017b). Plating for growth assays was only done at a maximum of five strains per plate to keep cells in the central portion of the plate, due to noticed position effects on toxicity, likely from evaporation, a known and observed characteristic of MCHM being its volatility (Phetxumphou *et al.*, 2016; Sain *et al.*, 2015). Furthermore, plates were used within a day to minimize any evaporation of MCHM from the media that would affect concentrations. Concentrations are noted in figure legends. When glutathione was used to attempt to rescue sensitive phenotypes, the concentration on plates was 10mM or 100mM, as noted. Oxidized glutathione was used at a concentration of 5mM corresponding to its structure as a dimer of glutathione. Ammonium sulfate was supplemented at a concentration of 10mM. Viability assays were carried out for 1.5 to 24 hr while kept in log-phase for concentrations indicated in figures.

### Flow cytometry

BY4741 cells were grown to saturation overnight and returned to mid-log phase. Cells were then diluted to a starting OD_600_ of 0.3 in biological triplicate in YPD media containing 550ppm MCHM. Cells were then harvested at the indicated time. Timepoints longer than 90 min required dilutions into fresh treatment media two times over the 12-hour experiment to maintain log-phase. The harvesting procedure involved taking 3mL of culture and fixing and preparing cells as previously published (Haase & Reed 2002). For cell cycle analysis, cell pellets were fixed in 70% ethanol overnight at 4°. Fixed cells were pelleted and resuspended in 0.5mL of a 2mg/mL RNAse A solution (50mM Tris pH 8.0, 15mM NaCl boiled for 15 min and cooled to room temperature) for a 12-hour incubation at 37°. Cells were then incubated in 0.2mL of a protease solution (5mg/mL pepsin, 4.5µl/mL HCl) for 20 min at 37°. Finally, samples were stored in 0.5mL of 50mM Tris pH 7.5 at 4° for up to a week before undergoing flow cytometric analysis. Immediately prior to flow cytometry, samples were shortly sonicated at low power on a Branson model SSE-1 Sonifier for six intervals of one-second bursts, and 50µl of each sample was suspended in 1µM SYTOX Green. Samples were analyzed on a BD LSRFortessa using a FITC channel. Approximately 30,000 events were collected for analysis. FCS Express 5.0 software was used to analyze the DNA content of cells using a multi-cycle DNA histogram and incorporated DNA modeling Statistics. Models were compared via Chi-squared results and the SL S0 model selected for determining the portion of the population in S phase for all samples. All replicates and timepoints fell within good or fair confidence for cell cycle modeling and S phase confidence of the model. The model produced the values for the proportions of the cell population falling within the G1, S, and G2/M phases used for analysis.

For measurement of ROS, live cells were pelleted then suspended in 200μl of 50mM DHE in phosphate-buffered saline (PBS). The dyed cultures were incubated at 30° for 20 min and washed with PBS. A positive control sample of BY4741 cells was treated with 25mM H_2_O_2_ for 1.5 hr. The DHE dyed samples were then analyzed within 2 hr of harvesting on a BD LSRFortessa using preset propidium iodide detection defaults. Approximately 30,000 events were collected per sample for downstream analysis. A High ROS subpopulation range was determined by gating events in downstream analysis at a DHE fluorescence level of 350. This value gave all three biological BY4741 wildtype strain replicates treated as the H_2_O_2_ positive controls approximately 95% of their cells falling above this value (94.69–96.86% actual percentages of cells). The wildtype and *vma3* cells, both untreated and treated with MCHM, that fell above a fluorescence level of 350 were considered to be part of a High ROS subpopulation. A one-tailed *t*-test was used to determine significance of increases in subpopulation percentages for each strain.

### Genetic screen

The genetic screen of the BY4742 haploid knockout collection was performed via the phenotyping of serial dilution growth on solid media by adapting a previous screen technique (Bae *et al.*, 2017). In short, frozen 96-well microtiter plates of the knockout collection were thawed and inoculated via pinning into 96-well growth chambers containing YPD in biological triplicate. Chambers were grown at 30° for 2 days until all wells were saturated. Each well was then serially diluted into 96-well microtiter plates three times at 20-fold concentrations, for three wells at 20-fold, 400-fold, and 8000-fold dilution from saturated. Diluted plates were then pinned onto large YPD solid media plates with or without 300ppm MCHM. Plates were visually scored for growth at 2-3 days, comparing control, and treated plates for each replicate. Knockout strains showing decreased growth of at least one spot, unaccounted for by decreased growth rate in general on control media, were recorded as screen “hits”. There was occasional variability in growth between replicates, so knockouts that did not replicate sensitive in all three trials filtered out.

### RNAseq

The transcriptomic dataset for this study was produced previously and published with full raw data output and a partial analysis (Gallagher *et al.*, 2020). The raw data are available at GSE, accession number GSE129898 (https://www.ncbi.nlm.nih.gov/geo/query/acc.cgi?acc=GSE129898). A partial reanalysis for this paper included comparisons of specifically strains of the BY4741 untreated control and the BY4741 MCHM treated cells. The library prep, transcript quantification with salmon (v0.9.1), bioinformatics pipeline, and differential analysis with DESeq2(1.18.1) were all performed exactly as in the previous study (Gallagher *et al.*, 2020). GO Term analysis was performed with DAVID bioinformatic database as below for this study.

### Comet assay

DNA damage resulting from MCHM exposure was quantitated using comet tails as previously described (Azevedo *et al.*, 2011; Oliveira & Johansson 2012). After incubation in MCHM for 30 min or hydrogen peroxide for 45 min (Heck *et al.*, 2010; Rowe *et al.*, 2008) as a positive control, single cells were solidified in 1.5% low-melting agarose. The cell walls were digested using 80 μl of 2mg/ ml zymolyase to create spheroplasts and immobilized on a glass slide. After cells were then lysed with 10 mM Tris HCl pH 10, 30 mM NaOH, 1 M NaCl, 50 mM EDTA and 0.05% w/v laurylsarcosine, they were rinsed three times with TAE, and nucleic acid was separated electrophoresis through the gel with 14V for 10 min. The gels were then neutralized Tris-HCl pH 7.4, and subsequently fixed with 76% and then 96% ethanol solutions to fix the DNA in place. DNA was stained with ethidium bromide for 20 min and then rinsed in water. The DNA fragments formed a tail from the rest of the DNA that has remained intact (the head). Images for each sample 17-42 ‘comets’ were captured with a Nikon A1R confocal microscope and measured via pixel intensity and distance (μm) of the DNA migration through the gel using TriTek CometScore 2.0.0.38. Statistical analysis was performed on tail length measurements using a one-way ANOVA and Tukey’s HSD test in base package of R version 3.6.3 (aov and TukeyHSD commands).

### GO Term analysis

The knockout screen gene list and the RNAseq differential analysis were each analyzed further using the GO Term function of the DAVID bioinformatic database at https://david.ncifcrf.gov (Huang *et al.*, 2009a, 2009b). Default settings were used, and no optional steps were included. Gene lists were searched for all three ontologies, though process and function were determined to show the most informative results and included in the main figures. Full results are available in supplemental files 1-3.

### Data availability

Raw data for transcriptome analysis are available at GSE, accession number GSE129898 (https://www.ncbi.nlm.nih.gov/geo/query/acc.cgi?acc=GSE129898). GO Term analysis results are available in supplemental files 1-3. The full gene lists of genetic screen hits, significantly upregulated genes, and significantly downregulated genes are available as Tables S1-S3. Haploid BY4741 and BY4742 knockout collection strains and YJM789 *aro1* knockout are available by request. The plasmids and/or resulting strains used in the *TAT2* overexpression analysis are available by request, or available commercially from DHARMACON. Crude MCHM used as the main reagent in the experiments was a gift from the Eastman Chemical Company, but a limited supply is available upon request. Supplemental material available at figshare: https://doi.org/10.25387/g3.13146365.

## Results

### Determination of S. cerevisiae sensitivity to MCHM treatment

**Figure 1:**
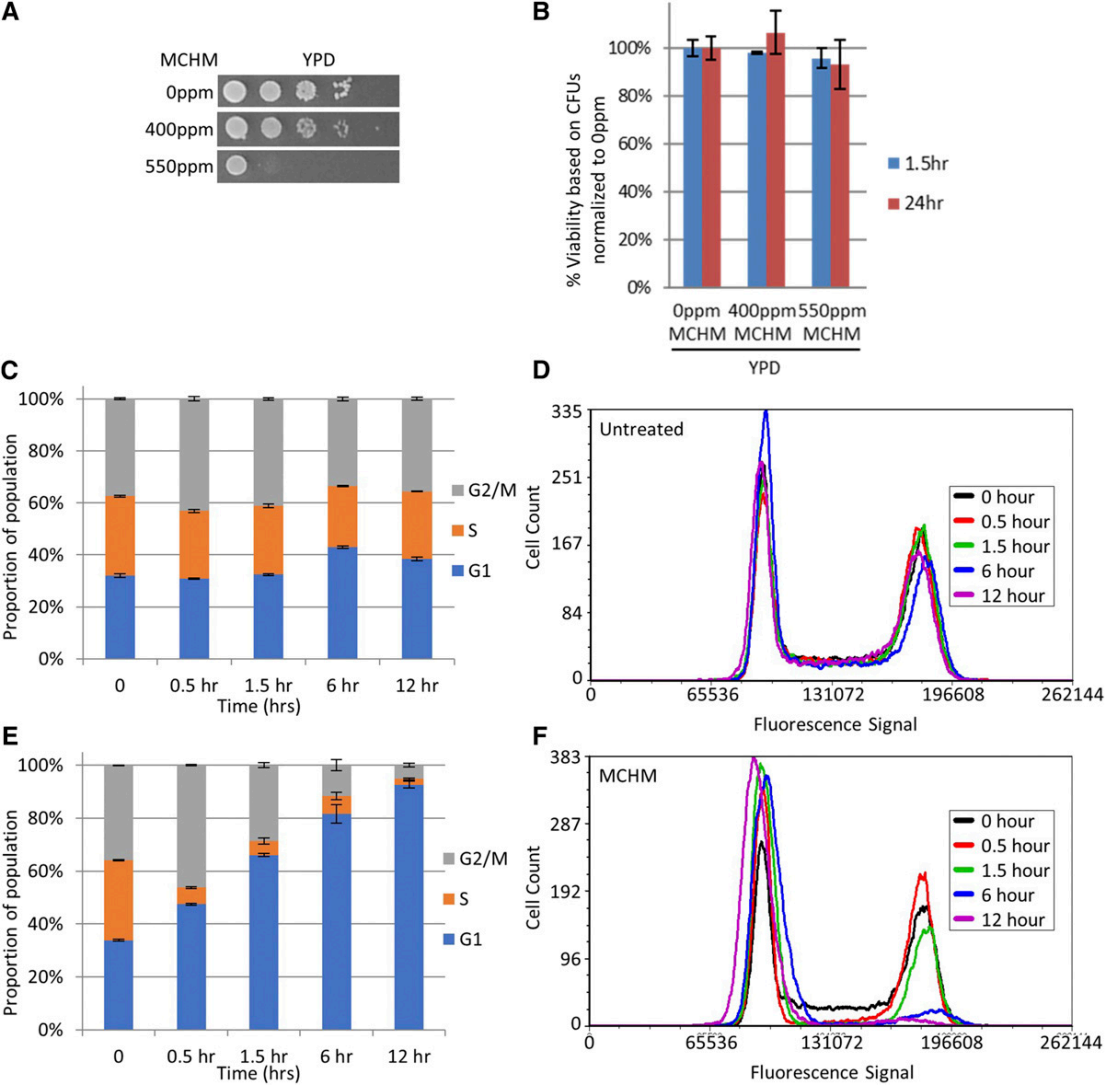
*Growth and viability of yeast under MCHM treatment*. A. Growth of 10-fold dilutions of yeast on YPD media with and without MCHM treatment. BY4741 strain is derived from the S288c background. B. Acute viability assay of the MCHM sensitive strain BY4741 treated with MCHM for 1.5 hr and 24 hr. Colony-forming units for three biological replicates were averaged for each treatment then normalized to media without MCHM treatment. In YPD yeast were exposed to 400ppm and 550ppm crude MCHM. C. Proportion of log-phase BY4741 in G1, S, or G2/M phases of the cell cycle based on flow cytometric analysis grown in YPD. D. Histograms of DNA content in BY4741 population grown in YPD shown in part C, as based on fluorescence intensity of sytox green. E. The proportion of log-phase BY4741 in G1, S, or G2/M phases of the cell cycle based on flow cytometric analysis grown in YPD with 550ppm MCHM. F. Histograms of DNA content of BY4741 population grown in YPD treated with MCHM shown in part E, as based on fluorescence intensity of sytox green. All samples were analyzed for 0, 0.5, 1.5, 6, and 12-hour time points to monitor cell cycle changes over time.

*S. cerevisiae* were treated with a range of MCHM levels to determine minimal concentrations that reduced growth in rich media (YPD). The levels were initially titrated, and approximately 400ppm MCHM inhibited the growth of BY4741 yeast (Figure 1A). The concentrations used on yeast in these growth assays (400-600ppm, 2.8-4.2mM) were approximately 100 times greater than the highest levels recorded in the water distribution system of Kanawha County, WV when monitoring began the day after the spill (Whelton *et al.*, 2014). While the growth assay phenotype itself requires a relatively high dosage of the chemical, the clear phenotype of decreased growth of yeast at this concentration is informative to the design of experiments that exploit the many genomic resources of the yeast. Similar chemical assays of toxic chemicals, such as hydrogen peroxide, use a comparable range of doses (0.5-5mM) to exploit the yeast growth phenotype as a measurement for cellular changes, including the original papers establishing the transcriptional programming of the yeast environmental stress response (0.3mM H_2_O_2_)(Azevedo *et al.*, 2011; Gasch *et al.*, 2000).

### Decreased growth of yeast treated With MCHM is not due to large-scale cell death

To assess whether MCHM inhibited growth by decreasing cellular viability or by arresting growth, acute and chronic viability assays were completed (Figure 1B). Mid-log phase yeast were acutely exposed for 90 min and chronically exposed for 24 hr to the 550ppm MCHM. Yeast were then washed and plated onto YPD with no MCHM. After growing plates for two days, there was little if any reduction in colony-forming units with the YPD-MCHM treated yeast.

To determine if the reduction in growth is due to cell cycle arrest, we analyzed MCHM treated yeast by flow cytometry (Figure 1C-1F). Untreated cellular populations showed an asynchronous pattern, for which each of the three phases had upwards of 20% of the total cellular population (Figure 1C and 1D). However, within 30 min, the MCHM treated samples showed that less than 10% of the population was in S phase (Figure 1E and 1F). The cells treated with MCHM appear to arrest almost immediately in G1 phase, leading to a nearly instantaneous loss of S phase population as no new cells leave G1. Over the course of the 12-hour experiment, the population in G2 also decreased to less than 10% of cells (Figure 1E and 1F). MCHM treated yeast failed to grow because they arrested in G1, not because of decreased viability.

### Genetic screen reveals cellular pathways and components required for MCHM tolerance

The cell cycle arrest of MCHM treated yeast was hypothesized to be attributable to environmental stress response programming, as many stressors initiate arrest as the cells attempt to ameliorate and recover from damage caused (Gasch *et al.*, 2000). In order to test this hypothesis, we collected genomic datasets to analyze which genes were functionally required for MCHM tolerance in mutant strains and which were differentially expressed in wildtype cells. The nearly 5000 strains of the haploid BY4742 knockout collection were tested for growth phenotypes on 300ppm MCHM in a genetic screen (Supplemental Figure 1). The results revealed that 329 genes were required for the tolerance to MCHM treatment (Supplemental Table 1). Several individually important environmental stress response genes were revealed by the screen. Two genes of note for their role in the control of Msn2/4-regulated stress responses were *MCK1* and *MSN5*. Deletions of these two genes have been shown previously to affect the ability of Msn2 to control stress response programming (Gutin *et al.*, 2019).

**Figure 2:**
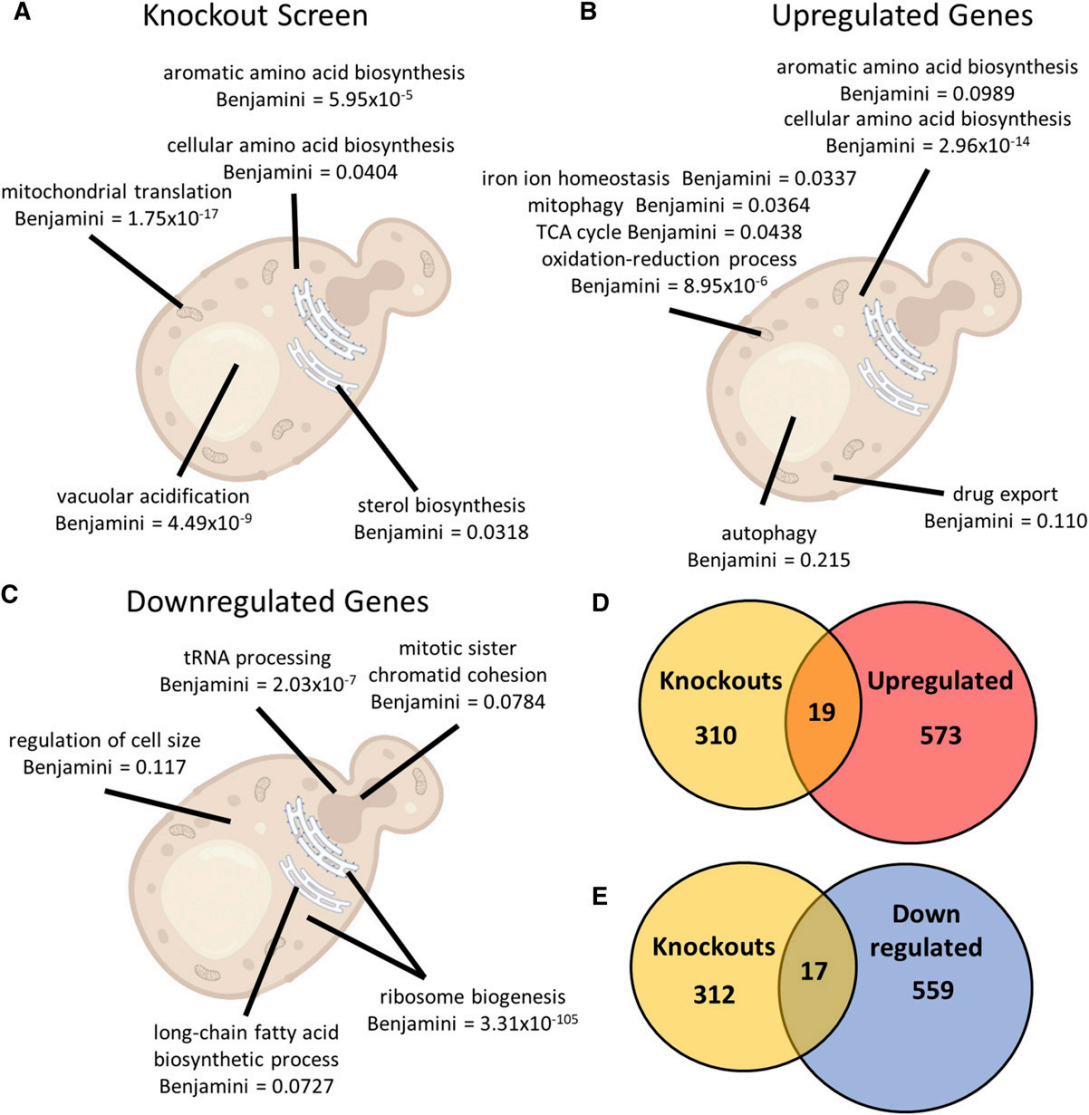
*Genetic screen of knockout collection and RNAseq of BY4741 gene lists*. A. Select top biological process GO Terms for the list of 329 genes found to be hits in the genetic screen of BY4742 knockout collection. Benjamini-Hochberg adjusted p-values reported by DAVID software are shown for each term. B. Select top biological process GO Terms for the 592 significantly upregulated genes from BY4741 treated with MCHM *vs.* untreated YPD. FDR is shown for each term. C. Select top biological process GO Terms for the 572 significantly downregulated genes from BY4741 treated with MCHM *vs.* untreated YPD. FDR is shown for each term. D. Venn diagram of the overlap between the 329 genes from the genetic screen and the upregulated genes. E. Venn diagram of the overlap between the 329 genes from the genetic screen and the downregulated genes.

GO term analysis of the results of the screen pointed to mitochondrial translation as the most enriched subset of yeast genes (Figure 2A). This particular GO term agrees well with previously published data showing that petite yeast strains are sensitive to MCHM (Pupo *et al.*, 2019a). Mitochondrial function is important for dealing with many stressors, especially those involved in reactive oxygen species production. The next most enriched gene subset was the components of the vacuolar ATPase (v-ATPase). This transporter maintains the acidity of the vacuole by pumping H+ ions across the vacuolar membrane. The homeostatic processes that use this ion gradient are evidence that MCHM may be causing ROS stress(N. Nelson & Harvey 1999; Nishi & Forgac 2002).

The next most enriched GO terms were for amino acid biosynthesis, especially aromatic amino acid biosynthesis. As the screen was performed on YPD media, it was surprising that these biosynthetic genes, including early precursor producing enzymes (*ARO1* and *ARO3*) and nearly the entirety of the tryptophan biosynthetic pathway (*TRP2**-**5*), were required when presumably excess amino acids were available from the media.

### MCHM treatment significantly affects the transcription of large portions of the yeast genome

Transcriptomic analysis of the BY4741 strain treated with MCHM also supported the hypothesis that the environmental stress response was activated with exposure to MCHM. There were 592 significantly upregulated genes in response to MCHM (Supplemental Table 2 and Figure 2B), while there were 576 genes significantly downregulated (Supplemental Table 3 and Figure 2C). These lists of genes were analyzed for GO terms and several of the terms were consistent with the activation of the ESR (Figure 2B-C). The upregulated GO terms for drug export, autophagy, and mitophagy point to ESR mechanisms for the removal of toxic substances from the cell and the reapportionment of cellular resources to overcome and adapt to a stressor. The upregulated terms related to amino acid biosynthesis and mitochondrial function (TCA cycle, iron ion homeostasis) were consistent with the genetic screen GO term analysis. The mitochondrial overlap between the datasets is likely due to the importance of ROS stress response, though the importance of the amino acid pathways as mentioned above, especially considering the cells are growing in rich media, is unclear.

The clearest indication for the central role of ESR activation by MCHM exposure was the downregulated GO terms. Ribosome biogenesis and related functions (other ribosomal terms were also significant as seen in Supplemental Table 3 but omitted from the figure for legibility) were the primary GO terms found in this dataset. They are also classic pathways downregulated in the ESR (Gasch *et al.*, 2000). Most ribosomal biogenesis protein genes are essential and do not appear in the haploid knockout collection used in the genetic screen, with 81% of the 79 ribosomal protein genes essential for growth in standard conditions (Steffen *et al.*, 2012). Therefore, the transcriptomic data were necessary supporting evidence for ESR involvement in MCHM response in combination with the stress gene and amino acid biosynthesis knockouts appearing in the screen. Other terms from the downregulated genes, including fatty acid biosynthesis and regulation of cell size, are also likely related to the observed cell cycle arrest and the reapportionment of energy and resources required for overcoming stressors.

### Combined genomic datasets implicate nutrient starvation

The genomic datasets individually revealed pathways involved in MCHM response, such as ESR activation from the transcriptome and vATPase and mitochondrial mutant sensitivity in the genetic screen. We decided to compare the genetic screen and transcriptome datasets to identify shared genes that might be especially important to MCHM response. The upregulated genes found in the knockout screen included those involved in mitochondrial function and ROS stress responses (Figure 2D and Table 1). *GGC1* and *TGL2* encode mitochondrial proteins involved in GTP/GDP transport and triacylglycerol lipase activity at the mitochondria respectively (Ham *et al.*, 2010; Vozza *et al.*, 2004). The *GGC1* knockout in particular produces petite yeast which are known to be sensitive to MCHM (Pupo *et al.*, 2019a; Vozza *et al.*, 2004). The *POS5* gene, identified originally for conferring peroxide sensitivity, is a mitochondrial protein that acts as an NADH kinase (Krems *et al.*, 1995; Strand *et al.*, 2003). It is required for maintenance of mitochondrial DNA, the loss of which results in the aforementioned petite yeast, as well as detoxification of ROS in the mitochondria (Strand *et al.*, 2003). The presence of these genes in both datasets points to the importance of mitochondrial function in the response to MCHM stress, most likely due to ROS production.

**Table 1:** List of genes overlapping between the genetic screen and upregulated or downregulated RNAseq results, as shown in Figure 2. Genes are organized, with subheadings, to highlight functions relevant to environmental stress responses

Overlap of Screen and Upregulated Genes	Overlap of Screen and Downregulated Genes
Aromatic Amino Acid Biosynthesis:	Aromatic Amino Acid Synthesis/Transport:
*ARO1*	*PRS1*
*ARO3*	*PRS3*
*TRP2*	*TAT1*
*TRP3*	
*TRP4*	RNA Processing/Ribosome Biogenesis:
*TRP5*	*MRT4*
	*NRS1*
Amino Acid Biosynthesis:	*RNR1*
*ILV6*	*RPA1*
*LYS2*	*TSR2*
*MET22*	
	Translation:
Mitochondrial Localization:	*BUD27*
*GGC1*	*FUN12*
*POS5*	
*TGL2*	Protein Chaperone:
	*YDJ1*
ABC Multi-drug Transporter:	
*SNQ2*	Transcriptional Regulator of Stress Response:
	*MOT3*
Transcription of rDNA:	
*RRN10*	Ion Transporter/Metal Homeostasis:
	*PHO84*
Miscellaneous Function:	
*MOG1*	Miscellaneous Function:
*XYL2*	*BUD30*
*VVS1 (YBR241C)*	*GUA1*
*TVS1 (YCR061W)*	*PRM7*
*YHI9*	*SAM1*

The other major insight of the combined screen and upregulation datasets is the presence of several amino acid biosynthetic genes. Many of these genes fall under the control of the general amino acid activator *Gcn4* (Braus 1991). Several single genes from pathways for isoleucine/valine (*ILV6*), methionine (*MET22*), and lysine (*LYS2*) were found in the overlap. Furthermore, the aromatic amino acid biosynthetic genes *ARO1* and *ARO3*, as well as four out of five tryptophan biosynthetic genes (*TRP2**, **TRP3**, **TRP4*, and *TRP5*), were found in both datasets. As tryptophan pathways in particular, but also tyrosine, have previously been implicated in the response to multiple types of stress including nutrient starvation, pH, metal ions, SDS, and DNA damaging chemicals, the precise role of *TRP* genes in MCHM tolerance remains to be elucidated (Godin *et al.*, 2016; González *et al.*, 2008; Schroeder & Ikui 2019).

The downregulated dataset overlaps with the screen also revealed genes involved in nutrient homeostasis (Figure 2E). *PRS1*, *PRS3*, and *TAT1* provide more evidence for the significance of tryptophan and amino acid metabolism in MCHM sensitivity. *PRS1* and *PRS3* encode 5-phospho-ribosyl-1(alpha)-pyrophosphate (PRPP) synthetase genes which are necessary for the production of purines and pyrimidines, and histidine and tryptophan amino acids (A. T. Carter *et al.*, 1997; Carter *et al.* 1994; Hernando *et al.*, 1999). While there are five paralog PRPP synthetases in yeast (*Prs* *1*-*5*), different multimeric complexes of the paralogous proteins are likely involved in producing wildtype levels of PRPP. Disruptions in *PRS1* and *PRS3* have the largest reduction in PRPP pools for nucleotide and amino acid production (Hernando *et al.*, 1999). The protein product of the *TAT1* gene is an amino acid transporter, characterized originally for its high-affinity import of tyrosine, but also shown to be a low-affinity transporter of tryptophan (Schmidt *et al.*, 1994). The combined screen and transcriptomic profiles indicated that MCHM may cause yeast cells to undergo nutrient starvation, possibly nitrogen specifically, despite growing in rich media. Cells may be responding by initiating cell cycle arrest and reapportioning nitrogen resources until recovered.

**Figure 3:**
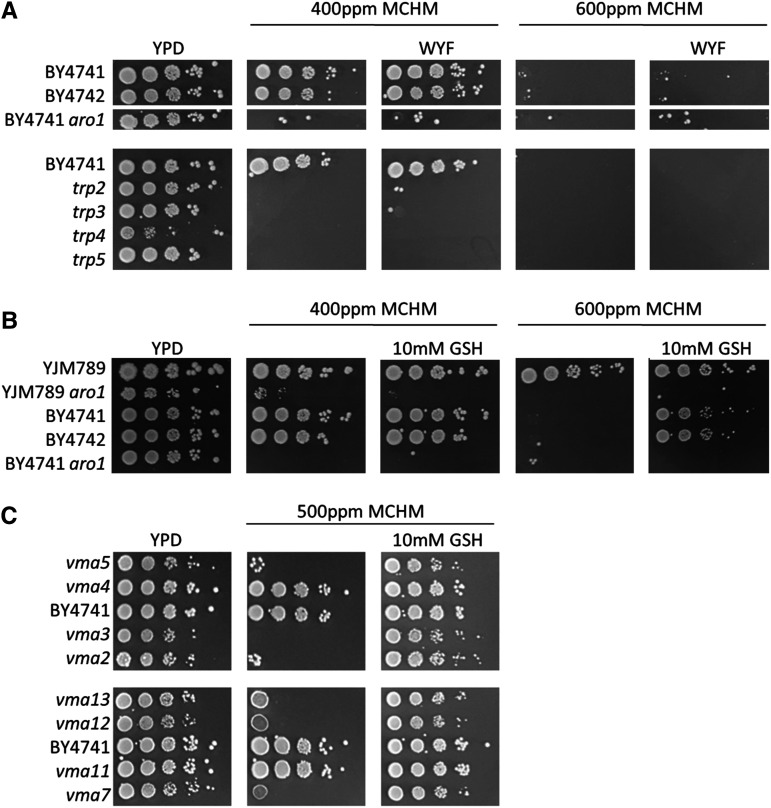
*Sensitive mutants from gene lists cotreated with possible rescuing chemicals* A. 10-fold serial dilution growth assay of knockouts of aromatic amino acid biosynthesis genes found in the genetic screen. Strains were cotreated with MCHM and aromatic amino acids Tryptophan (W), Tyrosine (Y), and Phenylalanine (F) in rich media, testing the extra supplementation of these amino acids beyond normal levels in YPD. B. 10-fold serial dilution growth assay of wildtype strains and knockout of aromatic amino acid biosynthetic gene *ARO1* with and without cotreatment of 10mM antioxidant glutathione (GSH) and MCHM. C. 10-fold serial dilution growth assay of knockouts in the vacuolar acidification pathway as found in the genetic screen. Strains were cotreated with and without 10mM antioxidant GSH and MCHM.

The importance of the aromatic amino acid synthetic genes in MCHM tolerance was investigated by additional supplementation of tryptophan, tyrosine, and phenylalanine in rich media containing MCHM. This was insufficient to rescue the growth of either wildtype or pathway gene knockout strains in MCHM (Figure 3A). It is possible that the excess tryptophan was not sufficiently imported into the cells based on the sorting of transporters such as Tat2 to the vacuole instead of the membrane (Schroeder & Ikui 2019). This is plausible as ergosterol levels in the cell affect this sorting preference, and MCHM is known to alter sterol levels in the cell (Pupo *et al.*, 2019b; Umebayashi & Nakano 2003). Overexpression of *TAT2* using a multicopy tiling plasmid did not rescue growth on MCHM with or without supplemental aromatic amino acids (Supplemental Figure 2). The levels of several amino acids increase with MCHM treatment, including the levels of tyrosine (Pupo *et al.*, 2019b). Unfortunately, the levels of tryptophan were not measured by GC-MS, so it is unknown if levels change with MCHM treatment. Yeast cells seem to be experiencing a nutrient starvation signal upon exposure, reacting by increasing production of amino acids in a way that is required according to knockouts from the screen, and yet failing to adapt to the chemical, based on the presence of excess amino acids alone.

### Uncharacterized ORFs both upregulated by and required for MCHM responses

Of note in the combined datasets is the requirement of *YBR241c* for tolerance to MCHM (Table 1). Ybr241c is a paralog of the vacuolar sorting protein, Vps73, and is localized to the vacuolar membrane (Matsumoto *et al.*, 2013). Acidification of the vacuole is critical for metabolizing chemicals such as MCHM and Ybr241c relocalizes to the cytoplasm when acidification of the vacuole is blocked (Matsumoto *et al.*, 2013). Ybr241c also physically interacts with Gtt1, a glutathione transferase of vacuolar proteins (Chandel *et al.*, 2016; Yu *et al.*, 2008). Like Vps73, Ybr241c shares homology with other sugar transporters such as Hxt1-17 and related proteins. As Ybr241 is a v acuolar protein v ital for s tress response, we named it Vvs1. Ycr061w localizes to cytoplasmic puncta that do not appear to be vacuoles but instead vesicles (Huh *et al.*, 2003; Tkach *et al.*, 2012). Ycr061w contains 10-11 predicted transmembrane domains (Weill *et al.*, 2019). As Ycr061w is a t ransmembrane protein v ital for s tress response, we named it Tvs1.

### Growth rescue by glutathione

As mitochondrial processes and iron ion homeostasis were found to be significant results in the genomic datasets, we further explored the role of reactive oxygen species in MCHM-induced stress. Glutathione serves as an antioxidant for the cell, particularly in the mitochondria and ER, and is also important in iron-sulfur homeostasis (Bulteau *et al.*, 2012). It was hypothesized that treatment with glutathione may rescue ROS stress created by exposure to MCHM, so yeast were co-treated with the chemicals. Glutathione was sufficient to rescue the growth of wildtype yeast, even at extremely high doses of 600ppm (Figure 3B). Tryptophan levels are important for resistance to DNA damaging agents (Godin *et al.*, 2016). However, glutathione was not able to rescue the sensitivity of the *aro1* mutant, so the unknown non-amino acid role in MCHM tolerance of the aromatic amino acid biosynthetic genes does not seem to be related to an ROS stress mechanism. The sensitivity of the *aro1* knockout mutant was replicated in a divergent yeast strain background, YJM789 (Figure 3B), to control for genetic background specific sensitivity.

The yeast vacuolar ATPase was the second most significant GO term from the genetic screen dataset. The v-ATPase is responsible for acidifying the vacuole of yeast cells by hydrolyzing ATP in order to pump hydrogen ions into the vacuole, creating an electrochemical gradient with multiple homeostatic roles (Deranieh *et al.*, 2015; H. Nelson & Nelson 1990; N. Nelson & Harvey 1999; Nishi & Forgac 2002; Ohya *et al.*, 1991). *vma* mutants show at least three sensitivity phenotypes that may be related to MCHM’s effects, including metal ion homeostasis, pH sensitivity, and oxidative stress sensitivity. To test if oxidative stress may be responsible for the sensitivity of *vma* mutants, we co-treated these mutants with MCHM and glutathione (Figure 3C). The observed growth rescue with glutathione treatment supports the hypothesis that the v-ATPase is required to provide a robust level of ROS protection yeast cells need to survive MCHM.

The antioxidant glutathione is a tripeptide of glycine, glutamate, and cysteine, and therefore, it is also potentially a source of nitrogen for cells. This complicates the interpretation of its role as an antioxidant source in rescue assays, given the role of nutrient deprivation upon exposure to MCHM. We tested if glutathione was acting solely as an antioxidant by treating the *glr1* knockout mutant with MCHM and oxidized glutathione. Glr1 is the glutathione reductase required to convert oxidized glutathione to reduced glutathione that can act as an antioxidant (Couto *et al.*, 2016). The oxidized glutathione was able to rescue the wildtype BY4742 strain and *glr1* mutant as well as reduced glutathione (Supplemental Figure 3). As the *glr1* mutant was rescued, the role of glutathione as antioxidant is called into question. We also treated cells with ammonium sulfate to test rescue by a general nitrogen source supplement, but the combination with MCHM was surprisingly toxic to cells (Supplemental Figure 3). As supplementation by aromatic amino acids, another nitrogen source, also failed to rescue growth, it is clear that if glutathione is acting as a nitrogen source instead of an antioxidant, it is unique among nitrogen supplements in its ability to alleviate the MCHM growth defect in cells.

### Biochemical assays for the presence of ROS in MCHM treated cells

**Figure 4:**
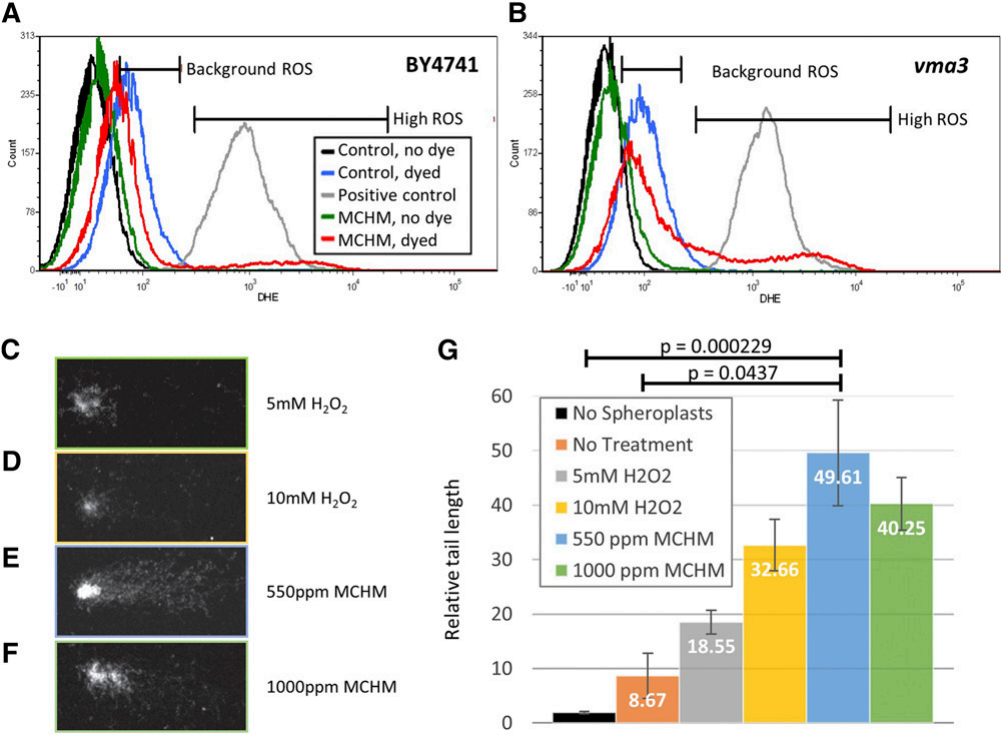
*Flow cytometry and comet assay of yeast strains reveals the production of Reactive Oxygen Species and DNA damage*. Levels of ROS in strains of yeast exposed to MCHM, based on fluorescence of the ROS-reactive dye DHE. Yeast were incubated for 12 hr with or without MCHM then stained with DHE for 20 min before cells were sorted using flow cytometry. Hydrogen peroxide treatment for 1.5 hr was used as a positive control to generate ROS in control cells (gray line). Background ROS of untreated yeast in blue measures endogenous ROS compared to unstained yeast with no DHE (black). MCHM treated yeast are in green while MCHM treated yeast stained with DHE are in red. Markers were inserted on the histogram to indicate a range of fluorescence corresponding to background or high ROS based on peaks for untreated control cells. Histograms are for the strains A. BY4741, and B. *vma3* knockout mutant. C-F. Examples of cells visualized by microscopy for the comet assay. Cells underwent treatment prior to spheroplasting, electrophoresis on a slide, and visualization via microscopy and ethidium bromide staining. Lengths of tails were measured with TriTek CometScore 2.0.0.38 software. The samples shown are examples of the following treatments C. 5mM H_2_O_2_, D. 10mM H_2_O_2_, E. 550ppm MCHM, and F. 1000ppm MCHM. G. Graph of tail lengths of cells determined via comet assay.

As genomic datasets and mutant growth assays had implicated ROS production as an effect of MCHM treatment, we attempted to directly detect ROS inside the cell. The dye dihydroethidium (DHE) reacts with intracellular ROS and then creates a fluorescent signal. We dyed yeast treated with MCHM with DHE, then detected the changes in fluorescent signal in the populations via flow cytometry. The flow cytometry results revealed an increase in fluorescence for a subpopulation of wildtype cells (Figure 4A). Within the genetic clonal population of MCHM treated cells represented by the red curve, phenotypic heterogeneity existed. The majority of MCHM treated cells did not have an increase in ROS levels, in contrast to hydrogen peroxide treated yeast (gray line). However, a subpopulation of the red curve, as seen in the portion of the curve under the marker labeled High ROS, did have increased ROS. The High ROS subpopulation of cells increased from 2.96 to 7.82% of the total cellular population in the BY4741 strain (Supplemental Figure 4). It is a known phenomenon that clonal populations in liquid culture can show phenotypic heterogeneity, specifically with respect to ROS sensitivity (Sumner *et al.*, 2003). This may explain why there is little reduction in the viability of BY4741 cells seen in Figure 1B. If the cells that successfully arrest their cell cycle were able to adapt and minimize their ROS production to avoid MCHM-induced death, they may appear as the large subpopulation with a lower ROS signal in the data. The cells that failed to do so may represent both the few cells that die in the viability assay, as well as those appearing as the small peak in the High ROS range. This assay revealed the first direct evidence that MCHM treatment produces ROS in the cell, in agreement with the indirect evidence originally seen in the sensitivity of *vma* mutants, glutathione rescue, and previous studies involving petite yeast (Pupo *et al.*, 2019a).

While the effects of MCHM on yeast point to an increase in ROS stress for the cell, wildtype cells seem to be robust enough, on average, to limit this stress, while mutants in certain pathways cannot. To test this hypothesis, we also performed the DHE assay on one of the most sensitive knockout mutant strains, *vma3*, which previously showed rescued growth with glutathione treatment. This mutant strain showed a similar pattern of DHE fluorescence when treated with MCHM, with the appearance of a High ROS population peak (Figure 4B). The major difference from the wildtype was an increase in the size of the High ROS population peak. The High ROS population in the *vma3* strain rose from 1.08 to 22.19% of all cells (Supplemental Figure 4). The mutant strain also appeared to show a right-shift of the background ROS populations as well. This may indicate *vma* mutants have higher ROS levels in unstressed conditions. Therefore, these strains have an innate sensitivity to MCHM due to their inability to maintain ROS homeostasis. We hypothesize that robust cells were able to detect the increased ROS caused by MCHM stress, then adapt to this by arresting growth and limiting their background ROS production.

### MCHM treatment causes DNA damage to wildtype yeast cells

We also questioned whether the ROS produced in MCHM treated cells may cause DNA damage, a known complication in ROS stressed cells (reviewed in Ayer *et al.*, 2014; Brennan *et al.*, 1994; Lafleur & Retèl 1993; Perrone *et al.*, 2008; Temple *et al.*, 2005). To address this question, a comet assay was performed to directly measure damage. This assay uses microscopy to measure the length of “tails”, resembling comets, released from spheroplasted and lysed cells, and migrated across agarose covered slides via electrophoresis (Figure 4C-F). Longer tails indicate DNA fragments, presumably from damage resulting in double-stranded breaks. Yeast cells treated with MCHM concentrations of 550 ppm showed significantly longer comet tails as compared to no treatment and no spheroplast controls (Tukey’s test p adj = 0.0437 and 0.000229 respectively) and approached significance as compared to 5mM H_2_O_2_ positive controls (p adj = 0.0655). This is the first direct evidence of DNA damage from MCHM treatment, confirming implications of data such as the ROS assay, cell cycle arrest, and other stress response data in previous studies (Lan *et al.*, 2015).

## Discussion

**Figure 5:**
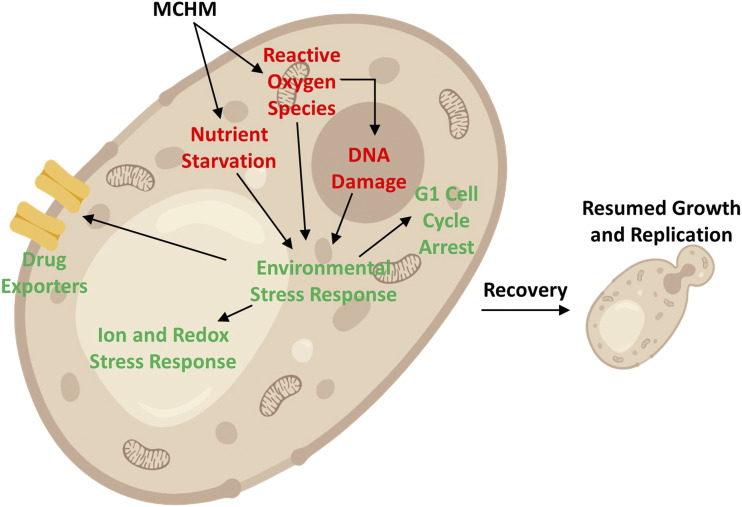
*Model of response and tolerance of yeast cells to MCHM treatment*. Diagram of yeast cell showing MCHM entering cell and causing ROS, DNA damage, and possible nutrient starvation. These signals are shown triggering environmental stress response leading to cellular responses that lead to recovery and resumption of replication.

The evidence presented here supports a model where MCHM activates the general environmental stress response programming of the yeast cell via Msn2/4 transcription factors, including specific responses consistent with ROS stress and nutrient starvation. Cell cycle arrest begins within 30 min of treatment, likely due to the initial signaling of nutrient starvation. These signals inactivate growth mechanisms soon after the cell is exposed, but before ROS accumulate. The ROS stress accumulates over six to twelve hours and the asynchronous population of cells continues to arrest in G1 during this time period. The long duration of the G1 arrest in MCHM treated cells is similar to that seen in nutrient starvation (Gasch *et al.*, 2000; Gasch & Werner-Washburne 2002). It is in contrast to other stresses such as oxidative stress and heat shock, which are more transient, with growth resuming after a short acclimation period to the stress. After a long acclimation period, robust strains such as wildtype were able to manage the stress, recover, and return to proliferation (Figure 5) at dosage levels significantly higher than those found in the Elk River spill. In this model, several signals initiate multiple stress responses, including nutrient starvation, reactive oxygen species, and DNA damage itself, but the nutrient starvation accounts for the most likely primary stress controlling the ESR and G1 arrest.

Nonetheless, there is direct evidence of ROS production and DNA damage, as well as rescue by treatment with glutathione which may be acting as either an antioxidant or a nitrogen source. Other data have previously indicated that petite yeast, known to be sensitive to ROS, are also sensitive to MCHM, and this agrees with the sensitivity of the mitochondrial translation mutants revealed in the genetic screen data of this study (Pupo *et al.*, 2019a). The vacuolar ATPase mutants, which are sensitive to metal and pH stress, as well as ROS, were one of the other most enriched GO terms from the screen. This study, combined with previous data, points to MCHM as a source of perturbation for metal ions and ROS (Lan *et al.*, 2015; Pupo *et al.*, 2019a).

The list of overlapping genes from the screen and the upregulated datasets included *SNQ2*, an ABC multidrug transporter associated with the removal of foreign substances from the cell (Figure 2D) (Decottignies *et al.*, 1995; Decottignies & Goffeau 1997; Rogers *et al.*, 2001; Servos *et al.*, 1993). While there are several such transporters (and related transcription factors) found in the transcriptomics, this is the only one found in the screen to be required for the tolerance of MCHM. We hypothesize that this transporter is responsible for the removal of MCHM or a related toxic byproduct from the cell. Future work on MCHM tolerance should include exploration of the relative roles of different ABC transporters in export of the chemical.

Another possible avenue for study is control of *Gcn4* and its related genes in the nutrient starvation signal detected during MCHM exposure. As previously noted, the amino acid biosynthetic genes *ILV6**, **MET22**, **LYS2**, **ARO1**, **ARO3**, **TRP2**, **TRP3**, **TRP4*, and *TRP5* are all under *Gcn4* control and are required for and upregulated in MCHM tolerance (Figure 2D and Table 1). The screen overlap with the downregulated dataset also includes several *Gcn4*-related genes (Figure 2E and Table 1). *BUD27* is related to translation initiation and may be involved in the expression of genes controlled by *Gcn4* (Deplazes *et al.*, 2009; Gstaiger *et al.*, 2003; Mirón-García *et al.*, 2013). *GUA1* is negatively regulated by nutrient starvation and its mutants have been shown to be impaired in *GCN4* translation (Escobar-Henriques *et al.*, 2003; Escobar-Henriques & Daignan-Fornier 2001; Iglesias-Gato *et al.*, 2011). *PRM7* also contains *Gcn4* binding elements, connecting it to control by this transcriptional activator (Schuldiner *et al.*, 1998). *GCN4* was not upregulated upon MCHM treatment at the transcriptional level. However, it is also controlled translationally, so it could have been upregulated at the protein level (Hinnebusch 1997; Meimoun *et al.*, 2000). It was also not required for tolerance in the genetic screen despite the importance the amino acid biosynthetic genes. Exploration of the background levels of expression of these genes in the *gcn4* knockout mutant could reveal if that is sufficient to perform their required functions in MCHM resistance.

Evidence of nutrient starvation is supported by the overlapping genomic datasets, as well as previous work which revealed that several amino acid levels increase in the cell (Pupo *et al.*, 2019b). Glutathione, in both its reduced and oxidized forms, were able to rescue growth, indicating that it may be acting as a nitrogen source instead of an antioxidant. However, treatment with excess aromatic amino acids nor general nitrogen sources such as ammonium sulfate could rescue growth. The cell’s programmed response to nutrient starvation is initiated by the cell to respond to MCHM, as wildtype cells upregulate the cellular and aromatic amino acid biosynthetic pathways. Furthermore, mutants lacking genes in these pathways, those in aromatic amino acids and tryptophan, in particular, are more susceptible to MCHM than wildtype cells, even in rich media with excess aromatic amino acids available.

The major question remains why the aromatic amino acid biosynthesis genes, including all but one of the tryptophan pathway genes, are required for resistance to the chemical. Supplementation with excess tryptophan in YPD did not rescue growth on MCHM, but it is not clear if this supplementation increased intracellular levels of tryptophan. It is possible the sorting of the transporter Tat2 to the plasma membrane was insufficient to import excess tryptophan. Furthermore, GC-MS failed to measure intracellular levels of tryptophan. While overexpression of *TAT2* was previously sufficient to rescue SDS sensitivity in *mck1* knockout mutant cells(Schroeder & Ikui 2019), a multicopy plasmid expressing the *TAT2* region of chromosome XV was not able to rescue growth on MCHM. Another hypothesis is that the biosynthetic genes themselves are important for signaling recovery from stress so that cells may resume proliferation, regardless of the actual levels of aromatic amino acid products related to the pathway. The *trp1**-**5* mutants are sensitive to rapamycin (González *et al.*, 2008), so it is possibly a recovery phenotype. The chemical glutathione can rescue MCHM treatment in wildtype cells, but it cannot do so in these mutants. Whether glutathione is ameliorating ROS stress or providing a specific nitrogen supplement when it rescues wildtype cells, it cannot do the same for these mutants. They may be required to perform a signaling role in the recovery after stress acclimation. Another possibility is that there is a separate source of stress besides nutrient availability or ROS that the genes are required to address, such as production or conversion of other small molecules. It would be useful to determine if there are other stressors for which these genes are required, regardless of amino acid levels, to address the possibility that this is specific to MCHM exposure.

The Elk River MCHM spill revealed that there are many things we do not know about the industrial chemicals that we live among. This and other studies since have revealed that levels of the chemical may not be acutely toxic, but there are immense and still only partially characterized effects on the biochemical pathways that control eukaryotic metabolism. This chemical causes the production of reactive oxygen species and DNA damage in yeast and alters the transcriptome and metabolome in extensive ways. While the effects on possible nutrient starvation and associated amino acid biosynthesis are not fully understood, cells with robust homeostatic processes are generally able to activate the environmental stress responses programmed in their genes to recover from MCHM exposure and resume proliferation.

## References

[bib1] Arndt K., and Fink G. R., 1986 GCN4 protein, a positive transcription factor in yeast, binds general control promoters at all 5′ TGACTC 3′ sequences. Proc. Natl. Acad. Sci. USA 83: 8516–8520. 10.1073/pnas.83.22.8516 3464968 PMC386961

[bib2] Ayer A., Gourlay C. W., and Dawes I. W., 2014 Cellular redox homeostasis, reactive oxygen species and replicative ageing in Saccharomyces cerevisiae. FEMS Yeast Res. 14: 60–72. 10.1111/1567-1364.12114 24164795

[bib3] Azevedo F., Marques F., Fokt H., Oliveira R., Orn Johansson B., 2011 Measuring oxidative DNA damage and DNA repair using the yeast comet assay. Yeast 28: 55–61. 10.1002/yea.1820 20824890

[bib4] Bae N. S., Seberg A. P., Carroll L. P., and Swanson M. J., 2017 Identification of Genes in *Saccharomyces cerevisiae* that Are Haploinsufficient for Overcoming Amino Acid Starvation. G3 (Bethesda) 7: 1061–1084. 10.1534/g3.116.037416 28209762 PMC5386856

[bib5] Brachmann C. B., Davies A., Cost G. J., Caputo E., Li J., 1998 Designer deletion strains derived from Saccharomyces cerevisiae S288C: a useful set of strains and plasmids for PCR-mediated gene disruption and other applications. Yeast 14: 115–132. 10.1002/(SICI)1097-0061(19980130)14:2<115::AID-YEA204>3.0.CO;2-2 9483801

[bib6] Braus G. H., 1991 Aromatic amino acid biosynthesis in the yeast Saccharomyces cerevisiae: A model system for the regulation of a eukaryotic biosynthetic pathway. Microbiol. Rev. 55: 349–370. 10.1128/MMBR.55.3.349-370.1991 1943992 PMC372824

[bib7] Brennan R. J., Swoboda B. E. P., and Schiestl R. H., 1994 Oxidative mutagens induce intrachromosomal recombination in yeast. Mutat. Res. Fundam. Mol. Mech. Mutagen. 308: 159–167. 10.1016/0027-5107(94)90151-1 7518043

[bib8] Bulteau A. L., Planamente S., Jornea L., Dur A., Lesuisse E., 2012 Changes in mitochondrial glutathione levels and protein thiol oxidation in ∆yfh1 yeast cells and the lymphoblasts of patients with Friedreich’s ataxia. Biochim. Biophys. Acta 1822: 212–225. 10.1016/j.bbadis.2011.11.003 22200491

[bib9] Capaldi A. P., Kaplan T., Liu Y., Habib N., Regev A., 2008 Structure and function of a transcriptional network activated by the MAPK Hog1. Nat. Genet. 40: 1300–1306. 10.1038/ng.235 18931682 PMC2825711

[bib10] Carter A. T., Beiche F., Hove-Jensen B., Narbad A., Barker P. J., 1997 PRS1 is a key member of the gene family encoding phosphoribosylpyrophosphate synthetase in Saccharomyces cerevisiae. Molecular and General Genetics MGG 254: 148–156. 10.1007/s004380050402 9108276

[bib11] Carter A. T., Narbad A., Pearson B. M., Beck K. F., Logghe M., 1994 Phosphoribosylpyrophosphate synthetase (PRS): A new gene family in Saccharomyces cerevisiae. Yeast 10: 1031–1044. 10.1002/yea.320100805 7992503

[bib12] Chandel A., Das K. K., and Bachhawat A. K., 2016 Glutathione depletion activates the yeast vacuolar transient receptor potential channel, Yvc1p, by reversible glutathionylation of specific cysteines. Mol. Biol. Cell 27: 3913–3925. 10.1091/mbc.E16-05-0281 27708136 PMC5170613

[bib13] Couto N., Wood J., and Barber J., 2016 The role of glutathione reductase and related enzymes on cellular redox homoeostasis network. Free Radic. Biol. Med. 95: 27–42. 10.1016/j.freeradbiomed.2016.02.028 26923386

[bib14] Cuozzo J. W., and Kaiser C. A., 1999 Competition between glutathione and protein thiols for disulphide-bond formation. Nat. Cell Biol. 1: 130–135. 10.1038/11047 10559898

[bib15] De Wever V., Reiter W., Ballarini A., Ammerer G., and Brocard C., 2005 A dual role for PP1 in shaping the Msn2-dependent transcriptional response to glucose starvation. EMBO J. 24: 4115–4123. 10.1038/sj.emboj.7600871 16281053 PMC1356311

[bib16] Decottignies A., and Goffeau A., 1997 Complete inventory of the yeast ABC proteins. Nat. Genet. 15: 137–145. 10.1038/ng0297-137 9020838

[bib17] Decottignies A., Lambert L., Catty P., Degand H., Epping E. A., 1995 Identification and Characterization of SNQ2, a New Multidrug ATP Binding Cassette Transporter of the Yeast Plasma Membrane. J. Biol. Chem. 270: 18150–18157. 10.1074/jbc.270.30.18150 7629127

[bib18] Deplazes A., Möckli N., Luke B., Auerbach D., and Peter M., 2009 Yeast Uri1p promotes translation initiation and may provide a link to cotranslational quality control. EMBO J. 28: 1429–1441. 10.1038/emboj.2009.98 19387492 PMC2688542

[bib19] Deranieh R. M., Shi Y., Tarsio M., Chen Y., McCaffery J. M., 2015 Perturbation of the vacuolar ATPase: A novel consequence of inositol depletion. J. Biol. Chem. 290: 27460–27472. 10.1074/jbc.M115.683706 26324718 PMC4645999

[bib20] Escobar-Henriques M., Collart M. A., and Daignan-Fornier B., 2003 Transcription Initiation of the Yeast IMD2 Gene Is Abolished in Response to Nutrient Limitation through a Sequence in Its Coding Region. Mol. Cell. Biol. 23: 6279–6290. 10.1128/MCB.23.17.6279-6290.2003 12917348 PMC180942

[bib21] Escobar-Henriques M., and Daignan-Fornier B., 2001 Transcriptional Regulation of the Yeast GMP Synthesis Pathway by Its End Products. J. Biol. Chem. 276: 1523–1530. 10.1074/jbc.M007926200 11035032

[bib22] Gallagher J. E. G., Ser S. L., Ayers M. C., Nassif C., and Pupo A., 2020 The Polymorphic PolyQ Tail Protein of the Mediator Complex, Med15, Regulates the Variable Response to Diverse Stresses. Int. J. Mol. Sci. 21: 1894 10.3390/ijms21051894 PMC7094212 32164312

[bib23] Garmendia-Torres C., Goldbeter A., and Jacquet M., 2007 Nucleocytoplasmic Oscillations of the Yeast Transcription Factor Msn2: Evidence for Periodic PKA Activation. Curr. Biol. 17: 1044–1049. 10.1016/j.cub.2007.05.032 17570669

[bib24] Gasch A. P., Spellman P. T., Kao C. M., Carmel-Harel O., Eisen M. B., 2000 Genomic Expression Programs in the Response of Yeast Cells to Environmental Changes. Mol. Biol. Cell 11: 4241–4257. 10.1091/mbc.11.12.4241 11102521 PMC15070

[bib25] Gasch A. P., and Werner-Washburne M., 2002 The genomics of yeast responses to environmental stress and starvation. Funct. Integr. Genomics 2: 181–192. 10.1007/s10142-002-0058-2 12192591

[bib26] Giaever G., Chu A. M., Ni L., Connelly C., Riles L., 2002 Functional profiling of the Saccharomyces cerevisiae genome. Nature 418: 387–391. 10.1038/nature00935 12140549

[bib27] Godin S. K., Lee A. G., Baird J. M., Herken B. W., and Bernstein K. A., 2016 Tryptophan biosynthesis is important for resistance to replicative stress in Saccharomyces cerevisiae. Yeast 33: 183–189. 10.1002/yea.3150 26804060 PMC4856547

[bib28] González A., Larroy C., Biosca J. A., and Ariño J., 2008 Use of the TRP1 auxotrophic marker for gene disruption and phenotypic analysis in yeast: A note of warning. FEMS Yeast Res. 8: 2–5. 10.1111/j.1567-1364.2007.00315.x 17892472

[bib29] Gorner W., Durchschlag E., Martinez-Pastor M. T., Estruch F., Ammerer G., 1998 Nuclear localization of the C2H2 zinc finger protein Msn2p is regulated by stress and protein kinase A activity. Genes Dev. 12: 586–597. 10.1101/gad.12.4.586 9472026 PMC316529

[bib30] Gstaiger M., Luke B., Hess D., Oakeley E. J., Wirbelauer C., 2003 Control of Nutrient-Sensitive Transcription Programs by the Unconventional Prefoldin URI. Science 302: 1208–1212. 10.1126/science.1088401 14615539

[bib31] Gutin J., Joseph‐Strauss D., Sadeh A., Shalom E., and Friedman N., 2019 Genetic screen of the yeast environmental stress response dynamics uncovers distinct regulatory phases. Mol. Syst. Biol. 15 10.15252/msb.20198939 PMC6711295 31464369

[bib32] Gutin J., Sadeh A., Rahat A., Aharoni A., and Friedman N., 2015 Condition-specific genetic interaction maps reveal crosstalk between the cAMP/PKA and the HOG MAPK pathways in the activation of the general stress response. Mol. Syst. Biol. 11: 829 10.15252/msb.20156451 26446933 PMC4631200

[bib33] Haase S. B., and Reed S. I., 2002 Improved Flow Cytometric Analysis of the Budding Yeast Cell Cycle. Cell Cycle 1: 117–121. 10.4161/cc.1.2.114 12429922

[bib34] Ham H. J., Rho H. J., Shin S. K., and Yoon H.-J., 2010 The TGL2 Gene of Saccharomyces cerevisiae Encodes an Active Acylglycerol Lipase Located in the Mitochondria. J. Biol. Chem. 285: 3005–3013. 10.1074/jbc.M109.046946 19959834 PMC2823425

[bib35] Heck D. E., Shakarjian M., Kim H. D., Laskin J. D., and Vetrano A. M., 2010 Mechanisms of oxidant generation by catalase. Ann. N. Y. Acad. Sci. 1203: 120–125. 10.1111/j.1749-6632.2010.05603.x 20716293 PMC4610122

[bib36] Hemenway C. S., Kara D., Cardenas M. E., Hiller M. A., Jones E. W., & I, J. H. (1995). *Require Calcineurin for Growth*. 844, 833–844. 10.1093/genetics/141.3.833 PMC1206848 8582630

[bib37] Hernando Y., Carter A. T., Parr A., Hove-Jensen B., and Schweizer M., 1999 Genetic Analysis and Enzyme Activity Suggest the Existence of More Than One Minimal Functional Unit Capable of Synthesizing Phosphoribosyl Pyrophosphate in Saccharomyces cerevisiae. J. Biol. Chem. 274: 12480–12487. 10.1074/jbc.274.18.12480 10212224

[bib38] Hiesinger P. R., Fayyazuddin A., Mehta S. Q., Rosenmund T., Schulze K. L., 2005 The v-ATPase V0 subunit a1 is required for a late step in synaptic vesicle exocytosis in Drosophila. Cell 121: 607–620. 10.1016/j.cell.2005.03.012 15907473 PMC3351201

[bib39] Hill D. E., Hope I. A., Macke J. P., and Struhl K., 1986 Saturation mutagenesis of the yeast his3 regulatory site: Requirements for transcriptional induction and for binding by GCN4 activator protein. Science 234: 451–457. 10.1126/science.3532321 3532321

[bib40] Hinnebusch A. G., 1997 Translational Regulation of Yeast GCN4. A window on factors that control initiator-trna binding to the ribosome. J. Biol. Chem. 272: 21661–21664. 10.1074/jbc.272.35.21661 9268289

[bib41] Hirata Y., Andoh T., Asahara T., and Kikuchi A., 2002 Yeast Glycogen Synthase Kinase-3 Activates Msn2p-dependent Transcription of Stress Responsive Genes. Mol. Biol. Cell 14: 302–312. 10.1091/mbc.e02-05-0247 PMC140246 12529445

[bib42] Huang D. W., Sherman B. T., and Lempicki R. A., 2009 a Systematic and integrative analysis of large gene lists using DAVID bioinformatics resources. Nat. Protoc. 4: 44–57. 10.1038/nprot.2008.211 19131956

[bib43] Huang D. W., Sherman B. T., and Lempicki R. A., 2009 b Bioinformatics enrichment tools: Paths toward the comprehensive functional analysis of large gene lists. Nucleic Acids Res. 37: 1–13. 10.1093/nar/gkn923 19033363 PMC2615629

[bib44] Huh W., Falvo J. V., Gerke L. C., Carroll A. S., Howson R. W., 2003 Global analysis of protein localization in budding yeast. Nature 425: 686–691. 10.1038/nature02026 14562095

[bib45] Iglesias-Gato D., Martín-Marcos P., Santos M. A., Hinnebusch A. G., and Tamame M., 2011 Guanine Nucleotide Pool Imbalance Impairs Multiple Steps of Protein Synthesis and Disrupts GCN4 Translational Control in Saccharomyces cerevisiae. Genetics 187: 105–122. 10.1534/genetics.110.122135 20980241 PMC3018310

[bib46] Jones G. M., Stalker J., Humphray S., West A., Cox T., 2008 A systematic library for comprehensive overexpression screens in Saccharomyces cerevisiae. Nat. Methods 5: 239–241. 10.1038/nmeth.1181 18246075

[bib47] Krems B., Charizanis C., and Entian K.-D., 1995 Mutants of Saccharomyces cerevisiae sensitive to oxidative and osmotic stress. Curr. Genet. 27: 427–434. 10.1007/BF00311211 7586028

[bib48] Lafleur M. V. M., & Retèl J. 1993 Contrasting effects of SH-compounds on oxidative DNA damage: Repair and increase of damage. Mutation Res. 295: 1–10. 10.1016/0921-8734(93)90006-O 7677924

[bib49] Lan J., Hu M., Gao C., Alshawabkeh A., & Gu A. Z. 2015 Toxicity Assessment of 4-Methyl-1-cyclohexanemethanol and Its Metabolites in Response to a Recent Chemical Spill in West Virginia, USA. Environ Sci. Technol. 49: 6284–6293. 10.1021/acs.est.5b00371 25961958 PMC6247413

[bib50] Li S. C., and Kane P. M., 2010 The yeast lysosome-like vacuole: endpoint and crossroads. Biochim. Biophys. Acta 1793: 650–663. 10.1016/j.bbamcr.2008.08.003 PMC2906225 18786576

[bib51] Martínez-Pastor M. T., Marchler G., Schüller C., Marchler-Bauer A., Ruis H., 1996 The Saccharomyces cerevisiae zinc finger proteins Msn2p and Msn4p are required for transcriptional induction through the stress response element (STRE). EMBO J. 15: 2227–2235. 10.1002/j.1460-2075.1996.tb00576.x 8641288 PMC450147

[bib52] Matsumoto R., Suzuki K., and Ohya Y., 2013 Organelle acidification is important for localisation of vacuolar proteins in Saccharomyces cerevisiae. Protoplasma 250: 1283–1293. 10.1007/s00709-013-0510-2 23708375

[bib53] Meimoun A., Holtzman T., Weissman Z., McBride H. J., Stillman D. J., 2000 Degradation of the Transcription Factor Gcn4 Requires the Kinase Pho85 and the SCFCDC4 Ubiquitin–Ligase Complex. Mol. Biol. Cell 11: 915–927. 10.1091/mbc.11.3.915 10712509 PMC14820

[bib54] Mirón-García M. C., Garrido-Godino A. I., García-Molinero V., Hernández-Torres F., Rodríguez-Navarro S., 2013 The Prefoldin Bud27 Mediates the Assembly of the Eukaryotic RNA Polymerases in an Rpb5-Dependent Manner. PLoS Genet. 9: e1003297 10.1371/journal.pgen.1003297 23459708 PMC3573130

[bib55] Nelson H., and Nelson N., 1990 Disruption of genes encoding subunits of yeast vacuolar H(+)-ATPase causes conditional lethality. Proc. Natl. Acad. Sci. USA 87: 3503–3507. 10.1073/pnas.87.9.3503 2139726 PMC53929

[bib56] Nelson N., and Harvey W. R., 1999 Vacuolar and plasma membrane proton-adenosinetriphosphatases. Physiol. Rev. 79: 361–385. 10.1152/physrev.1999.79.2.361 10221984

[bib57] Nishi T., and Forgac M., 2002 the Vacuolar (H+)-Atpases—Nature’S Most Versatile Proton Pumps. Nat. Rev. Mol. Cell Biol. 3: 94–103. 10.1038/nrm729 11836511

[bib58] Ohya Y., Umemoto N., Tanida I., Ohta A., Irda H., 1991 Calcium-sensitive cls mutants of Saccharomyces cerevisiae showing a Pet- phenotype are ascribable to defects of vacuolar membrane H+-ATPase activity. J. Biol. Chem. 266: 13971–13977. 1830311

[bib59] Oliphant A. R., Brandl C. J., and Struhl K., 1989 Defining the sequence specificity of DNA-binding proteins by selecting binding sites from random-sequence oligonucleotides: Analysis of yeast GCN4 protein. Mol. Cell. Biol. 9: 2944–2949. 10.1128/MCB.9.7.2944 2674675 PMC362762

[bib60] Oliveira R., & Johansson B. 2012 Quantitative DNA Damage and Repair Measurement with the Yeast Comet Assay. Methods Mol. Biol. 920: 101–109. 10.1007/978-1-61779-998-3_8 22941599

[bib61] Perrone G. G., Tan S.-X., and Dawes I. W., 2008 Reactive oxygen species and yeast apoptosis. *Biochimica et Biophysica Acta (BBA)* -. Molecular Cell Research 1783: 1354–1368. 10.1016/j.bbamcr.2008.01.023 18298957

[bib62] Phetxumphou K., Dietrich A. M., Shanaiah N., Smiley E., and Gallagher D. L., 2016 Subtleties of human exposure and response to chemical mixtures from spills. Environ. Pollut. 214: 618–626. 10.1016/j.envpol.2016.04.056 27131822

[bib63] Pupo A., Ayers M. C., Sherman Z. N., Vance R. J., Cumming J. R., 2019a MCHM Acts as a Hydrotrope, Altering the Balance of Metals in Yeast. Biol. Trace Elem. Res. 10.1007/s12011-019-01850-z PMC7150659 31392542

[bib64] Pupo A., Ku K. M., and Gallagher J. E. G., 2019b Effects of MCHM on yeast metabolism. PLoS One 14: e0223909 10.1371/journal.pone.0223909 31622418 PMC6797124

[bib65] Rogers B., Decottignies A., Kolaczkowski M., Carvajal E., Balzi E., 2001 The pleitropic drug ABC transporters from Saccharomyces cerevisiae. J. Mol. Microbiol. Biotechnol. 3: 207–214. 11321575

[bib66] Rong-Mullins X., Ravishankar A., McNeal K. A., Lonergan Z. R., Biega A. C., 2017a Genetic variation in Dip5, an amino acid permease, and Pdr5, a multiple drug transporter, regulates glyphosate resistance in S. cerevisiae. PLoS One 12: e0187522 10.1371/journal.pone.0187522 29155836 PMC5695762

[bib67] Rong-Mullins X., Winans M. J., Lee J. B., Lonergan Z. R., Pilolli V. A., 2017b Proteomic and genetic analysis of the response of S. Cerevisiae to soluble copper leads to improvement of antimicrobial function of cellulosic copper nanoparticles. Metallomics 9: 1304–1315. 10.1039/C7MT00147A 28869270 PMC5741080

[bib68] Rowe L. A., Degtyareva N., and Doetsch P. W., 2008 DNA damage-induced reactive oxygen species (ROS) stress response in Saccharomyces cerevisiae. Free Radic. Biol. Med. 45: 1167–1177. 10.1016/j.freeradbiomed.2008.07.018 18708137 PMC2643028

[bib69] Sain A. E., Dietrich A. M., Smiley E., and Gallagher D. L., 2015 Assessing human exposure and odor detection during showering with crude 4-(methylcyclohexyl)methanol (MCHM) contaminated drinking water. Sci. Total Environ. 538: 298–305. 10.1016/j.scitotenv.2015.08.050 26311585

[bib70] Sambade M., Alba M., Smardon A. M., West R. W., and Kane P. M., 2005 A genomic screen for yeast vacuolar membrane ATPase mutants. Genetics 170: 1539–1551. 10.1534/genetics.105.042812 15937126 PMC1365767

[bib71] Santhanam A., Hartley A., Düvel K., Broach J. R., and Garrett S., 2004 PP2A Phosphatase Activity Is Required for Stress and Tor Kinase Regulation of Yeast Stress Response Factor Msn2p. Eukaryot. Cell 3: 1261–1271. 10.1128/EC.3.5.1261-1271.2004 15470255 PMC522594

[bib72] Schmidt A., Hall M. N., and Koller A., 1994 Two FK506 resistance-conferring genes in Saccharomyces cerevisiae, TAT1 and TAT2, encode amino acid permeases mediating tyrosine and tryptophan uptake. Mol. Cell. Biol. 14: 6597–6606. 10.1128/MCB.14.10.6597 7523855 PMC359189

[bib73] Schroeder L., and Ikui A. E., 2019 Tryptophan confers resistance to SDS-associated cell membrane stress in Saccharomyces cerevisiae. PLoS One 14: e0199484 10.1371/journal.pone.0199484 30856175 PMC6411118

[bib74] Schuldiner O., Yanover C., and Benvenisty N., 1998 Computer analysis of the entire budding yeast genome for putative targets of the GCN4 transcription factor. Curr. Genet. 33: 16–20. 10.1007/s002940050303 9472075

[bib75] Sekito T., Sugimoto N., Ishimoto M., and Kawano-kawada M., 2014 Tributyltin induces cell cycle arrest at G1 phase in the yeast Saccharomyces cerevisiae. J. Toxicol. Sci. 39: 311–317. 10.2131/jts.39.311 24646713

[bib76] Servos J., Haase E., and Brendel M., 1993 Gene SNQ2 of Saccharomyces cerevislae, which confers resistance to 4-nitroquinoline-N-oxide and other chemicals, encodes a 169 kDa protein homologous to ATP-dependent permeases. Molecular and General Genetics MGG 236: 214–218. 10.1007/BF00277115 8437567

[bib77] Sikorski R. S., and Hieter P., 1989 A system of shuttle vectors and yeast host strains designed for efficient manipulation of DNA in Saccharomyces cerevisiae. Genetics 122: 19–27. 2659436 10.1093/genetics/122.1.19 PMC1203683

[bib78] Steffen K. K., McCormick M. A., Pham K. M., MacKay V. L., Delaney J. R., 2012 Ribosome Deficiency Protects Against ER Stress in Saccharomyces cerevisiae. Genetics 191: 107–118. 10.1534/genetics.111.136549 22377630 PMC3338253

[bib79] Strand M. K., Stuart G. R., Longley M. J., Graziewicz M. A., Dominick O. C., 2003 POS5 Gene of Saccharomyces cerevisiae Encodes a Mitochondrial NADH Kinase Required for Stability of Mitochondrial DNA. Eukaryot. Cell 2: 809–820. 10.1128/EC.2.4.809-820.2003 12912900 PMC178377

[bib80] Sumner E. R., Avery A. M., Houghton J. E., Robins R. A., & Avery S. V. (2003). Cell cycle- and age-dependent activation of Sod1p drives the formation of stress resistant cell subpopulations within clonal yeast cultures. Mol Microbiol. 50: 857–870. 10.1046/j.1365-2958.2003.03715.x 14617147

[bib81] Temple M. D., Perrone G. G., and Dawes I. W., 2005 Complex cellular responses to reactive oxygen species. Trends Cell Biol. 15: 319–326. 10.1016/j.tcb.2005.04.003 15953550

[bib82] Thomasson E. D., Scharman E., Fechter-Leggett E., Bixler D., Ibrahim S., 2017 Acute Health Effects After the Elk River Chemical Spill, West Virginia, January 2014. Public Health Rep. 132: 196–202. 10.1177/0033354917691257 28182515 PMC5349493

[bib83] Tkach J. M., Yimit A., Lee A. Y., Riffle M., Costanzo M., 2012 Dissecting DNA damage response pathways by analyzing protein localization and abundance changes during DNA replication stress. Nat. Cell Biol. 14: 966–976. 10.1038/ncb2549 22842922 PMC3434236

[bib84] Toledano M. B., Delaunay-Moisan A., Outten C. E., and Igbaria A., 2013 Functions and cellular compartmentation of the thioredoxin and glutathione pathways in yeast. Antioxid. Redox Signal. 18: 1699–1711. 10.1089/ars.2012.5033 23198979 PMC3771550

[bib85] Umebayashi K., and Nakano A., 2003 Ergosterol is required for targeting of tryptophan permease to the yeast plasma membrane. J. Cell Biol. 161: 1117–1131. 10.1083/jcb.200303088 12810702 PMC2172991

[bib86] Vozza A., Blanco E., Palmieri L., and Palmieri F., 2004 Identification of the Mitochondrial GTP/GDP Transporter in Saccharomyces cerevisiae. J. Biol. Chem. 279: 20850–20857. 10.1074/jbc.M313610200 14998997

[bib87] Weidhaas J. L., Dietrich A. M., DeYonker N. J., Ryan Dupont R., Foreman W. T., 2016 Enabling Science Support for Better Decision-Making when Responding to Chemical Spills. J. Environ. Qual. 45: 1490–1500. 10.2134/jeq2016.03.0090 27695739

[bib88] Weill U., Cohen N., Fadel A., Ben-Dor S., and Schuldiner M., 2019 Protein Topology Prediction Algorithms Systematically Investigated in the Yeast Saccharomyces cerevisiae. BioEssays 41: 1800252 10.1002/bies.201800252 PMC7611422 31297843

[bib89] *West Virginia Chemical Spill: Collective NTP Findings and Supporting Files*. (n.d.). National Toxicology Program U.S. Department of Health and Human Services.

[bib90] Whelton A. J., McMillan L. K., Connell M., Kelley K. M., Gill J. P., 2014 Residential Tap Water Contamination Following the Freedom Industries Chemical Spill: Perceptions, Water Quality, and Health Impacts. Environ. Sci. Technol. 49: 813–823. 10.1021/es5040969 25513829

[bib91] Yu H., Braun P., Yildirim M. A., Lemmens I., Venkatesan K., 2008 High-quality binary protein interaction map of the yeast interactome network. Science 322: 104–110. 10.1126/science.1158684 18719252 PMC2746753

